# The Association Between Smartphone Addiction/Overuse With Hand and Wrist Musculoskeletal Complaints, Saudi Arabia

**DOI:** 10.7759/cureus.48752

**Published:** 2023-11-13

**Authors:** Bassmh Abdullah A Al-Dhafer, Haidar A Alessa, Mohammed A Albesher, Muna F Alnaim, Sara K Albawardi, Maitham Albesher

**Affiliations:** 1 Orthopedic Surgery, King Faisal University, Al Ahsa, SAU; 2 College of Medicine, King Faisal University, Al Ahsa, SAU; 3 Plastic and Reconstructive Surgery, King Faisal University, Al Ahsa, SAU; 4 Plastic Surgery, King Faisal University, Al Ahsa, SAU; 5 Medicine and Surgery, Ministry of Health, Al Ahsa, SAU

**Keywords:** wirst pain, musculoskeletal hand pain, smartphone addiction, hand and wrist pain, carpal tunnel syndome

## Abstract

Introduction

Smartphones have integrated seamlessly into our daily lives in various aspects. When a smartphone is used frequently for communication or internet access, it becomes addictive, which increases the risk of musculoskeletal problems in the hand, wrist, and thumb.

Aim

This research aimed to examine if there is a connection between excessive smartphone use and discomfort in the thumb and wrist.

Subject and methods

This cross-sectional study was conducted among the general population of Saudi Arabia. A self-administered questionnaire translated into Arabic was distributed among the targeted population using an online survey. The questionnaire includes three sections, including socio-demographic data (e.g., age, gender, nationality, etc.), assessment of smartphone addiction by using the smartphone addiction scale short-version (SAS-SV), and assessment of wrist/hand pain using the patient-rated wrist and hand evaluation (PRWHE).

Results

Of the 3057 recruited participants, 1938 (63.4%) were females, and 3025 (99%) were aged between 18 and 65 years old. Perceived pain in the wrist or hand due to excessive usage of a smartphone was reported by 1728 (56.5%) of respondents. The overall mean SAS-SV score was 24.4 (SD 7.47) out of 50 points. The prevalence of smartphone addiction among the general population was 874 (28.6%). Increased SAS-SV and PRWHE scores were associated with respondents living outside Eastern region and using phones for 10 hours or more daily.

Conclusion

The prevalence of smartphone addiction in this study was 874 (28.6%), directly associated with wrist and hand pains. Musculoskeletal complaints due to smartphone addiction were more prevalent among respondents living outside Eastern Region who spent more time using their phones. A longitudinal study is required to establish the link between smartphone addiction and musculoskeletal complaints among the general population.

## Introduction

In this closely interconnected digital age, there has been a phenomenal rise in the number of people using smartphones for communication, entertainment, work, and study [1]. An individual with behavioral addiction cannot control harmful behaviors that originate from an impulse or urge. Compulsive shopping, internet addiction, eating disorders, and gambling are some examples of the various forms of behavioral addiction that are too frequent today [2]. In recent years, smartphone addiction or overuse has emerged as a significant global concern, influencing people of all ages and backgrounds, including students. The prevalence of smartphone addiction among university students in the Kingdom of Saudi Arabia increased dramatically between 2016 and 2019, from 19.1% to 60.3% [3,4]. Also, it has been discovered that smartphone addiction and overuse are linked to subsequent stress, sleep issues, behavioral changes, mood swings, and even depression. In comparison to the elder generation, students are more dependent on cellphones and may be more susceptible to smartphone addiction [5]. With smartphone addiction/overuse come various physical complaints, most notably musculoskeletal disorders such as pain in the neck, shoulders, hand/wrist, and elbows. This may be because of the static pressures placed on the cervical and upper extremity musculoskeletal systems from prolonged lousy posture while using a smartphone, such as a flexed neck, unsupported elbows, and repetitive thumb movements to scroll around the screen [6-9]. Previous studies aimed at examining the link between smartphone use and upper limb diseases among Saudi university students tended to focus on university students [10,11]. None of the prior studies have been conducted on the general population of all Saudi Arabian regions. There is a noticeable dearth of research examining this topic in the existing literature. As a result, this research aimed to examine if there is a connection between excessive smartphone use and discomfort in the thumb and wrist.

## Materials and methods

This study is a cross-sectional study that targeted the general population in Saudi Arabia to determine the association between smartphone addiction/overuse and hand and wrist musculoskeletal complaints. The study was conducted between May 2023 and October 2023. An online survey was created using Google Forms for data collection. The online survey was spread to the general population in each of the five regions of Saudi Arabia, respectively (central, eastern, northern, western, and southern regions), and people were encouraged and invited to participate. To reach the population from each region of the five regions and to enroll as many participants as possible, many data collectors were recruited from every region. Data was collected through an online self-administered questionnaire, where participants first consented to participate in the study before starting to fill out the questionnaire. The questionnaire included three sections. The first section asked about socio-demographic data, including age, gender, nationality, residency, region, education level, occupation, marital status, and smoking status. The second part was concerned with smartphone addiction or overuse by using the smartphone addiction scale short-version (SAS-SV) [12], which included time, concentrating, standing without a smartphone, feeling impatient, missing planned activities, and several other questions. The third section asked about wrist/hand pain using the patient-rated wrist and hand evaluation (PRWHE-A) [13]. Also, the participants were asked if there was any paresthesia, numbness, or weakness to give an idea if the patient might have a nerve injury. The study was presented to specialists in orthopedics for improvement and approval; it was first written in English and then translated into Arabic for it to be comprehensible for the targeted population. The Arabic version was examined by three different language experts, and the translation was approved after grammatical and linguistic modifications. After that, a pilot study was performed on a small group of people (15 people) to confirm a uniform understanding of the questions. 

Statistical analysis

The Smartphone Addiction Scale-Short Version (SAS‑SV) has been used to assess the smartphone addiction of the sample population, consisting of 10 out of 33 questions. The cutoff point to determine the level of smartphone addiction was 29 points, as recommended by Servidio et al. [14]. 

IBM Corp. Released 2019. IBM SPSS Statistics for Windows, Version 26.0. Armonk, NY: IBM Corp has been used to analyze the data for this project. Categorical variables were distributed and presented as numbers and percentages. Continuous variables were given and summarized as mean ± standard deviation and median (min-max). The association between SAS-SV and PRWHE in relation to the socio-demographic variables has been conducted using the Mann-Whitney Z-test as well as the Kruskal-Wallis H-test. Normality tests and statistical collinearity were performed using the Shapiro-Wilk test as well as the Kolmogorov-Sminov test. Both SAS-SV and PRWHE scores follow a non-normal distribution. Therefore, the non-parametric tests were applied. The Spearman correlation coefficient has been used to determine the correlation between the SAS-SV score and the PWRHE score. Statistical significance was set at the p-value <0.05 level.

Study population

The study’s subjects were all the general population of Saudi Arabia who consented to participate in this study during its period between May and October 2023 and met the inclusion criteria. The inclusion criteria consisted of being an adult aging 16 years and older, living in Saudi Arabia, and having consented to participate in the study. The exclusion criteria consisted of being younger than 16 years old, living outside of Saudi Arabia, and not consenting to participate in the study. The sampling technique that will be utilized is convenient random sampling, where the questionnaire is disseminated via social media platforms and the general population of Saudi Arabia is invited to participate through an online link. Sample size was calculated using the formula n = z2pq\d 2. With a confidence level of 95%, an estimated proportion of 50%, and a 5% level of precision. The minimum sample size was calculated to be 385. However, more participants and candidates were included to ensure the sufficiency and accuracy of the results.

## Results

Three thousand and fifty-seven participants met the inclusion criteria. The socio-demographic characteristics of participants are shown in Table 1. Nearly all 3025 (99%) were aged between 18 and 65 years old, with females being dominant (63.4%). Most of our respondents were Saudis, with 2777 (90.8%) and 1186 (38.8%) currently living in the Eastern Region. More than half (1664, 54.4%) were single, whereas 2376, 77.7%, had higher educational levels. Approximately 1278 (41.8%) were students. Respondents who were smokers constituted 439 (14.4%). A great proportion of respondents were right-handed. In addition, 1792 (58.6%) were using phones between 5 and 9 hours a day. 

**Table 1:** Socio-demographic characteristics of participants (n=3057)

Study variables	N (%)
Age group	
18 to 65 years	3025 (99.0%)
>65 years	32 (01.0%)
Gender	
Male	1119 (36.6%)
Female	1938 (63.4%)
Nationality	
Saudi	2777 (90.8%)
Non-Saudi	280 (09.2%)
Region	
Central Region	540 (17.7%)
Eastern Region	1186 (38.8%)
Northern Region	318 (10.4%)
Western Region	630 (20.6%)
Southern Region	383 (12.5%)
Marital status	
Single	1664 (54.4%)
Married	1297 (42.4%)
Divorced	64 (02.1%)
Widowed	32 (01.0%)
Educational level	
Low education	681 (22.3%)
High education	2376 (77.7%)
Occupational status	
Student	1278 (41.8%)
Employed	1023 (33.5%)
Retired	239 (07.8%)
Unemployed	499 (16.3%)
Self-employed	18 (0.60%)
Smoking status	
Smoker	439 (14.4%)
Non-smoker	2618 (85.6%)
Dominant hand	
Right hand	2756 (90.2%)
Left hand	301 (09.8%)
How much time do you use your phone daily?	
0 to 4 hours	735 (24.0%)
5 to 9 hours	1792 (58.6%)
≥10 hours	530 (17.3%)

In Table 2, the prevalence of participants who experienced pain in the wrist or hand due to phone use was 1728 (56.6%). Among those who experienced pain in the wrist or hand (n=1728), 386 (22.3%) of them had an operation in the mentioned musculoskeletal region. Most of those who experienced pain had pain in their right hand 1048 (60.6%), felt numbness 619 (35.8%), and had pain if they tried to put their thumb in the palm of their hand and tilt 1125 (65.1%).

**Table 2:** Prevalence of hand pain due to phone use (n=3057)

Variables	N (%)
Have you ever felt pain in your wrist or hand due to phone use?	
Yes	1728 (56.5%)
No	1329 (43.5%)
Have you had pain or an operation on your wrist/hand? ^(n=1728)^	
Yes	386 (22.3%)
No	1342 (77.7%)
Which hand has pain? ^(n=1728)^	
Right hand	1048 (60.6%)
Left hand	269 (15.6%)
Both	411 (23.8%)
Do you feel any numbness or weakness in the affected hand? ^(n=1728)^	
Numbness Only	619 (35.8%)
Weakness Only	356 (20.6%)
Numbness and Weakness	411 (23.8%)
None of them	342 (19.8%)
Is there pain if you put your thumb in the palm of your hand and then fist it and tilt it downward? ^(n=1728)^	
Yes	1125 (65.1%)
No	603 (34.9%)

In Table 3, the overall mean SAS-SV score was 24.4 (SD 7.47). Assessing PRWHE among respondents who experienced pain in their wrist/hand (n=1728) found that the mean score of the pain domain was 23.2 (SD 9.71), while in the function domain, the total mean score was 16.1 (SD 10.2). The overall mean PRWHE score was 39.2 (SD 18). 

**Table 3:** Descriptive statistics of the smartphone addiction scale, short version (SAS-SV), and patient-rated wrist and hand evaluation (PRWHE) (n = 3057)

Variables	Mean ± SD	Median	Min	Max
SAS-SV score	24.4 ± 7.47	24.0	8.00	40.0
PRWHE score ^(n=1728)^	39.2 ± 18.0	37.5	10.0	100
Pain score	23.2 ± 9.71	23.0	5.00	50.0
Function score	16.1 ± 10.2	13.5	5.00	50.0

**Figure 1:**
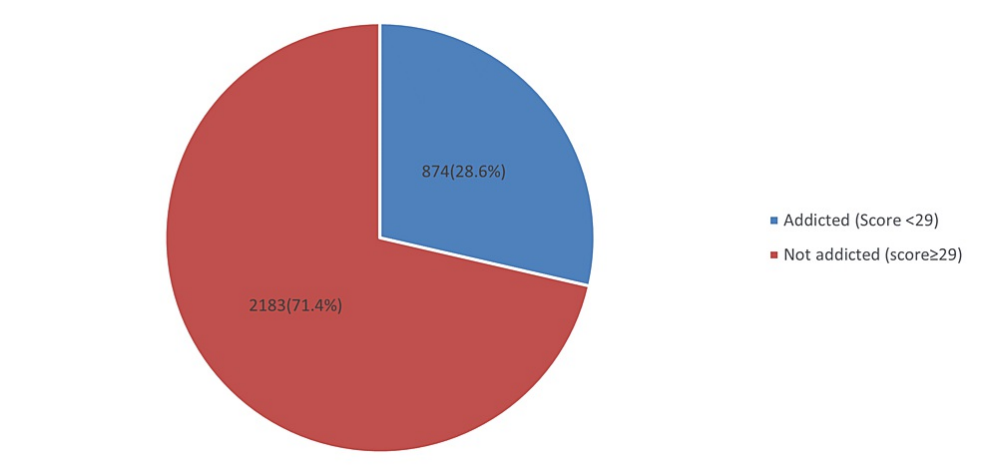
Prevalence of smartphone addiction according to SAS-SV criteria

In Figure 1, the prevalence of smartphone addiction among the general population based on SAS-SV criteria was 874 (28.6%), and the rest were not addicted 2183 (71.4%).

**Figure 2:**
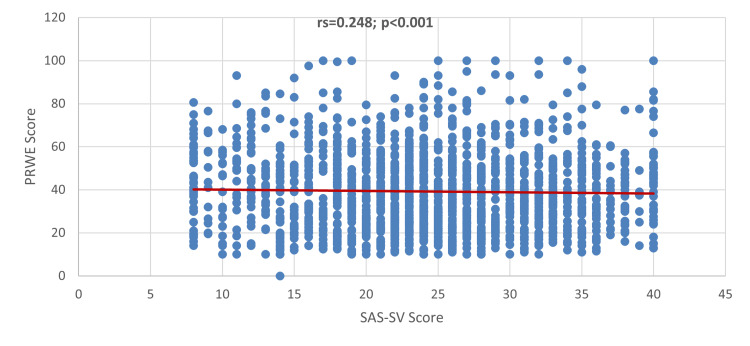
Correlation between SAS-SV score and PRWHE score

Figure 2 shows a significant positive correlation between the SAS-SV score and the PRWHE score (rs=0.248; p<0.001). 

When measuring the association between SAS-SV and PRWHE in terms of participants' socio-demographic characteristics (Table 4), it was found that a higher SAS-SV score was more associated among participants living outside the Eastern Region (Z=2.435; p=0.015), being unmarried (Z=7.456; p<0.001), being a student (H=65.080; p<0.001), being a smoker (Z=3.046; p=0.002), and using 10 hours or more of phone daily (H=374.78; p<0.001). We also observed that a higher PRWHE was more associated among participants living outside Eastern Region (Z=3.191; p=0.001), being married (Z=1.966; p=0.049), and daily use of a phone for 10 hours or more (H=17.369; p<0.001).

**Table 4:** Association between SAS-SV and PRWHE scores in relation to the socio-demographic characteristics of participants a P-value has been calculated using the Mann-Whitney Z-test. b P-value has been calculated using the Kruskal-Wallis H-test. ** Significant at the p<0.05 level.

Factor	SAS-SV ^(n=3057)^ Score (50) Mean ± SD	Z/H-test; P-value	PRWHE ^(n=1728)^ Score (100) Mean ± SD	Z/H-test; P-value
Gender ^a^				
Male	24.3 ± 7.47	0.022; 0.982	38.7 ± 16.5	0.131; 0.896
Female	24.4 ± 7.47		39.5 ± 18.7	
Nationality ^a^				
Saudi	24.4 ± 7.49	0.662; 0.508	39.3 ± 18.1	0.832; 0.406
Non-Saudi	24.6 ± 7.32		37.9 ± 16.8	
Region ^a^				
Inside Eastern Region	24.0 ± 7.32	2.435; 0.015 **	37.4 ± 17.4	3.191; 0.001 **
Outside Eastern Region	24.6 ± 7.55		40.3 ± 18.3	
Marital status				
Unmarried	25.3 ± 7.44	7.456; <0.001 **	38.4 ± 17.5	1.966; 0.049 **
Married	23.2 ± 7.33		40.4 ± 18.6	
Educational level ^a^				
Low education	23.8 ± 7.55	2.500; 0.012 **	40.3 ± 17.6	1.461; 0.144
High education	24.6 ± 7.44		38.9 ± 18.1	
Occupational status ^b^				
Student	25.7 ± 7.29	65.080; <0.001 **	38.2 ± 17.0	2.741; 0.254
Employed	23.8 ± 7.59		39.4 ± 18.0	
Unemployed	22.9 ± 7.24		40.6 ± 19.6	
Smoking status ^a^				
Smoker	25.3 ± 7.74	3.046; 0.002 **	40.6 ± 17.9	1.340; 0.180
Non-smoker	24.2 ± 7.41		38.9 ± 18.1	
Dominant hand ^a^				
Right hand	24.4 ± 7.44	0.634; 0.526	39.3 ± 18.0	0.803; 0.422
Left hand	24.5 ± 7.79		38.3 ± 18.0	
How much time do you use your phone daily? ^b^				
0 to 4 hours	20.6 ± 7.01	374.78; <0.001 **	37.8 ± 18.0	17.369; <0.001 **
5 to 9 hours	24.6 ± 6.90		38.3 ± 16.9	
≥10 hours	28.9 ± 7.28		43.3 ± 20.2	

## Discussion

This study investigated the link between smartphone addiction and hand and wrist musculoskeletal complaints. This study revealed a positive association between smartphone addiction and hand/wrist musculoskeletal pains. A positive but low degree of correlation was observed between the SAS-SV and PRWHE scores (rs=0.248; p<0.001), indicating that the increased time of smartphone device use will likely increase the risk of hand and wrist disabilities. This is almost consistent with the report by Shah and Sheth [1]. A study found that there was a moderately positive correlation between SAS and the neck disability index (NDI) and between SAS and the Cornell Hand Discomfort Questionnaire (CHDQ). This had also been observed in a study conducted by Baabdullah et al., wherein smartphone addiction was positively correlated with a high PRWHE score [10]. Other studies also found a significant association between smartphone addiction and musculoskeletal-related pains, such as the study done in Bangladesh [5], India [15], and Turkey [16]. Contradicting these reports, research conducted by Osailan [17] documented a weak but inverse correlation between smartphone usage duration versus hand grip and pinch grip strength.

According to the SAS-SV criteria, the prevalence of smartphone addiction in this study was 874 (28.6%) (mean score: 24.4; SD 7.47, out of 50 points). This report is higher than the study carried out by Alkhateeb et al. [3], with a smartphone addiction prevalence of 370 (19.1%) with an overall count of participants in 1941. In contrast, several studies documented a higher prevalence of smartphone addiction detected among the general population (60.3%) [4], medical students (256, 66.4%), with an overall number of individuals involved of 387, and university students (93, 55.3%) among the female participants in the study (185 participants)with total number of 367 participants [10,11]. The increasing prevalence of smartphone addiction is quite alarming yet uncontrollable. Hence, continuous efforts to educate smartphone users about the dangers posed by prolonged use of electronic devices are necessary. Highlighting the importance of following daily screen time usage may reduce smartphone addiction's prevalence and ultimately lead to a decreased prevalence of musculoskeletal complaints.

Data in our study suggest that living outside the Eastern Region, being unmarried, having a higher level of education, being a student, being a smoker, and increasing time spent on phone use were identified as the significant predictors of smartphone addiction. In a study done by Mustafaoglu et al. [15], they also found a significant correlation between the SAS score in terms of the duration of smartphone use in a typical day and the duration of owning a smartphone, while in a study by Alsalameh et al. [4], the level of the academic year was statistically associated with the level of smartphone addiction.

According to PRWHE criteria, the overall mean score was 39.2 (SD 18) out of 100 points, suggesting a low degree of musculoskeletal pain. Regarding PRWHE domains, the pain domain has a higher mean score (23 points out of 50 points) than the function domain (mean score: 16.1). Among the general public in Bangladesh [5], a significant proportion of respondents complained about neck pain (141, 43.3%), shoulder pain (136, 42.9%), and elbow pain (88, 27.9%) during prolonged smartphone usage, with a total of 326 participants. This has been observed in a study conducted in Turkey with a total of 249 participants [16], where 175 (70.3%), 171 (68.7%), and 164 (65.9%) of the respondents complained about pains in the upper back, wrists/hands, and neck that occurred during excessive use of smartphones.

Complaints of musculoskeletal disorders were more frequently seen among married respondents living outside the Eastern Region and spending more time using smartphones. According to the report of Sirajudeen et al. [11], female gender and participants with increased weight were significantly associated with the occurrence of musculoskeletal disorders. However, in a study by Khaled et al. [18], they found no significant association between disability scores in terms of gender and age. Our study also observed this, wherein PRWHE scores have no significant association with gender, nationality, education, occupation, smoking status, or the dominant hand (p>0.05).

Moreover, we noted that 1728 (56.5%) of our respondents complained of pain in the wrist and hand due to phone use, particularly in the right hand (1048) (60.6%), with feeling numbness (619) (35.8%). In a scenario among those who suffered musculoskeletal disorders, by putting the thumb in the palm of their hand, then fisting it, and then tilting the hand downward, approximately two-thirds (65.1%) suffered pain. This scenario further validates the connection between smartphone addiction and hand pain, which may be a short-term disability but could lead to a long-term one if neglected.

Limitation

The findings of this study were subjected to some limitations. First, the age group was not equally distributed. Hence, we cannot generalize the comparison between the younger and older age groups. Second, this data was collected using an online survey. Thus, some participants may not be truthful when answering the questionnaire. Lastly, being cross-sectional is prone to selection, information bias, and confounding. It does not measure cause and effect.

## Conclusions

More than one-quarter of the general population is addicted to smartphones, which are costly affect the wrist and hands. Smartphone addiction was widely prevalent among unmarried respondents with a higher education who were smokers and spent significant time using their phones. However, married respondents who lived outside the Eastern Region were more likely to complain about musculoskeletal disorders due to the prolonged use of smartphones than any other respondents. Excessive usage of smartphones is detrimental to health, both physically and psychologically. Therefore, healthy usage of electronic devices should be promoted to decrease the prevalence of smartphone-related musculoskeletal diseases.
